# Expansion of mesenchymal adipose-tissue derived stem cells in a stirred single-use bioreactor under low-serum conditions

**DOI:** 10.1186/1753-6561-7-S6-P2

**Published:** 2013-12-04

**Authors:** Carmen Schirmaier, Stephan C Kaiser, Valentin Jossen, Silke Brill, Frank Jüngerkes, Christian van den Bos, Dieter Eibl, Regine Eibl

**Affiliations:** 1 Zurich University of Applied Sciences, Institute of Biotechnology, Biochemical Engineering and Cell Cultivation Technique, 8820 Wädenswil, Switzerland; 2 Lonza Cologne GmbH, 50829 Cologne, Germany

## Background

The need for human mesenchymal stem cells (hMSCs) has increased enormously in recent years due to their important therapeutic potential. Efficient cell expansion is essential to providing clinically relevant cell numbers. Such cell quantities can be manufactured by means of scalable microcarrier (MC)-supported cultivations in stirred single-use bioreactors.

## Materials and methods

Preliminary tests in disposable-spinners (100 mL culture volume, Corning) were used to determine two suitable media and MC-types for serum reduced expansions (< 10%) of human adipose tissue-derived stem cells (hADSCs; passage 2, Lonza). Using such optimized media-MC-combinations, hADSCs expanded 30 to 40-fold, which compares well with expansion rates in planar culture. Based on computational fluid dynamics simulations and suspension analyses in spinners [1], optimal operating parameters were determined in a BIOSTAT^® ^UniVessel^® ^SU 2 L (2 L culture volume, Sartorius Stedim Biotech).

## Results

In subsequent batch tests with the BIOSTAT UniVessel^® ^SU 2 L, expansion rates of between 30 and 40-fold were reached and up to 4.4·10^8 ^cells with a cell viability exceeding 98% were harvested. Flow cytometry tests demonstrated typical marker profiles following cell expansion and harvest. A 40-fold expansion rate delivered a total of 1·10^10 ^cells in a first cultivation with the BIOSTAT^® ^CultiBag STR 50 L (35 L culture volume, Sartorius Stedim Biotech).

## Conclusions

In summary, the foundations for successfully expanding therapeutic stem cells in truly scalable systems have been laid. Strategies ensuring expansion rates between 60 and 70-fold and, thus, generating cell amounts over 10^10 ^are now in preparation.
